# Accumulation of Plastics and Trace Elements in the Mangrove Forests of Bima City Bay, Indonesia

**DOI:** 10.3390/plants12030462

**Published:** 2023-01-19

**Authors:** Hanna Moniuszko, Win Ariga Mansur Malonga, Piotr Koczoń, Sofie Thijs, Robert Popek, Arkadiusz Przybysz

**Affiliations:** 1 Section of Basic Research in Horticulture, Department of Plant Protection, Institute of Horticultural Sciences, Warsaw University of Life Sciences—SGGW (WULS—SGGW), Nowoursynowska 159, 02-776 Warsaw, Poland; 2 Department of Nature Resource Conservation, Sumbawa University of Technology, Olat Maras Street, Moyohulu District, Sumbawa Regency 84371, Indonesia; 3 Department of Chemistry, Institute of Horticultural Sciences, Warsaw University of Life Sciences—SGGW (WULS—SGGW), Nowoursynowska 159, 02-776 Warsaw, Poland; 4 Environmental Biology, Centre for Environmental Sciences, Hasselt University, Agoralaan Building D, 3590 Diepenbeek, Belgium

**Keywords:** absorption, adsorption, *Avicennia alba*, microplastic, nanoplastic, pneumatophores, polyamide, selective accumulation, soil sediments, vinylidene chloride

## Abstract

Pollution with microplastics (MPs), nanoplastics (NPs) and trace elements (TEs) remains a considerable threat for mangrove biomes due to their capability to capture pollutants suspended in the water. This study investigated the abundance and composition of plastics and TEs contained in the soil and pneumatophores of *Avicennia alba* sampled in experimental areas (hotel, market, river mouth, port, and rural areas) differentiated in anthropopressure, located in Bima Bay, Indonesia. Polymers were extracted and analyzed with the use of a modified sediment isolation method and Fourier transform infrared spectroscopy. Trace elements were detected by inductively coupled plasma optical emission spectrometry. The lowest and highest quantities of MPs in soil were recorded in rural and hotel areas, respectively. The rural site was characterized by distinct MP composition. The amounts of sediment-trapped MPs in the tested localities should be considered as high, and the recognized polymers partly corresponded with local human activity. Concentrations of seven plastic types found in plant tissues did not entirely reflect sediment pollution with nine types, suggesting a selective accumulation (particularly of polyamides and vinylidene chloride) and substance migration from other areas. Very low concentrations of non-biogenic TEs were observed, both in sediments and pneumatophores. The results highlight the relevance of environmental contamination with plastics.

## 1. Introduction

Coastal and marine ecosystems are continuously being threatened by eutrophication, the influx of toxic substances (including trace elements and plastic fragments), and acidification, originating mainly from land-based industry and agriculture, which generate c. 80% of the pollution load [1,2,3,4]. Indonesia has struggled with anthropogenic pressures on its coastal ecosystems in recent decades due to its increasing population proportionally contributing to the intensified contamination of the seashore zone [5].

Plastic is one of the main pollutants of coastal areas [5]. It is estimated to constitute up to 54% (by mass) of the anthropogenic waste released into the environment [6]. Besides the oceans and coastlines, plastics have been so far detected in rivers [7,8] and lakes [9], wherein they create a so-called ‘plastisphere’ [10]. The prevalence of plastics in the environment is a consequence of the global demographic explosion combined with the contemporary ‘single-use plastic culture’, mismanaged plastic waste and its low biodegradability [11,12,13]. The material undergoes fragmentation and due to aging, physical, chemical and biological forces, degrades into so-called microplastics (MPs). Microplastic is defined as a synthetic, solid particle or polymeric matrix of differentiated chemical composition, regular or irregular in shape, and size ranging from 1 μm to 5 mm. The further fragmentation results in nanoplastics (NPs; 1–100 nm) [14,15,16,17]. Their principal sources in water reservoirs are domestic wastewater effluents, sewage discharges, the plastic manufacturing industry and decomposition products of larger pieces [18]; therefore, their abundance is highly dependent on population density and the types of human activities [19].

Seashore areas are vulnerable to the dispersion of trace elements (TEs), which are also a consequence of anthropopressure [20]. Increasing TE inflows into coastal wetlands, rivers, lakes and bays has become a serious concern, posing threats to biodiversity and human health [21,22,23,24,25]. This type of contamination has been recognized, also along the Indonesian coast, in sediments surrounding spots with developed industry and mining, agriculture, and paint and battery factories as well as industrial waste disposal zones [22,26,27,28]. Trace elements are absorbed from the environment in ionic form via roots along with water and nutrients. The process is shaped mainly by the substrate acidity, amount of organic matter, presence of clay minerals and interactions between given elements [29].

Mangrove forests cover approximately 180,000 km^2^ of the Earth, accounting for 0.12% of the total land surface and 0.5% of the world’s coastal areas [30,31]. Mangroves are broad-leaved evergreen pioneer halophytes [32]. These plants are habitats, breeding grounds and food sources for a variety of animals, but also stabilize marine ecosystems [33]. They are key carbon reservoirs [31], regulate regional climate [34,35,36], reduce coastal erosion, encourage sediment deposition and link sea and fresh waters [37]. Mangroves vegetate in shallow, brackish to salty water and are influenced by the tidal current, which is a factor shaping the entry of nutrients and freshwater [37]. Such a changing habitat implies anatomical adaptations. *Avicennia* spp. trees have a few types of roots. Among them, aerial roots or pneumatophores are responsible for gas exchange in muddy substratum. These erect organs are covered by water and airtight, spongy periderm, except for the lenticels—crater-like holes enabling oxygen intake [38].

The mangrove biome is recognized worldwide as constantly threatened due to its proximity to urban and industrial zones that dump a wide range of pollutants with little or no treatment [39,40]. Mangrove thickets are natural filters for the contaminants that do not readily decompose, such as MPs [9,40,41,42,43], and thereby play an important role in the ecological purification of water [44,45]. The muddy mangrove sediment can contain up to eight times more MP than non-mangrove residues [42]. These plants are also considerable depositional stores of sediment-associated TEs due to their ability to control the redox conditions of diagenetic reactions and to enrich the sediments in organic matter that is capable of binding TEs [22]. The presence of TEs was also detected in *Avicennia* spp. plants [4,22,46]; however, the identified concentrations in plants were generally lower than in the sediment [22].

The highly negative influence of the plastisphere results not only from its ubiquity in the water environment and trophic chains, but also from its affinity to adsorb toxins (including TEs and organic compounds) and its provision of favorable conditions for bacterial biofilms harboring pathogenic and opportunistic bacteria [17,47,48,49]. These properties result from large, uneven and chemically active surfaces of plastic particles, acting as vectors that transfer adsorbed contaminants, among other materials, to the rhizosphere, thus increasing their concentrations [30,50]. The types and sizes of particles, given elements, physical conditions and microbial activity affect the adsorption/desorption of TEs on MPs [11,51]. Nonetheless, the observed TE concentrations on MP particles are several times higher than in the water column [47].

As outlined above, both plastics and TEs tend to deposit and accumulate in the mangrove forests. Therefore, in this study, we examined mangrove sediments as well as pneumatophores of *Avicennia alba* plants for the presence of plastic and trace elements. Our objective was the quantitative and qualitative analysis of plastic polymers and TEs. In order to assess the relation linking the anthropogenization and contamination levels, samples were collected in five mangrove zones, differentiated with regard to human activity (i.e., rural location, hotel area, marketplace, river mouth and port area), in the vicinity of Bima city, Indonesia.

## 2. Results

### 2.1. Salinity Levels and Length of the Pneumatophores

**Figure 1:**
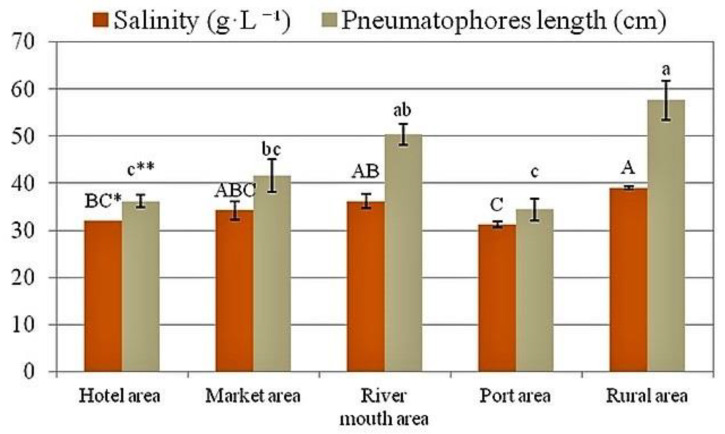
Salinity and length of pneumatophores from five mangrove areas in Bima City Bay. Data are mean ± SE, *n* = 5. * Capital letters indicate significant differences between salinity levels, ** Lowercase letters indicate significant differences between lengths of pneumatophores.

The lengths of pneumatophores from five experimental locations corresponded well with the salinity values (Figure 1). The higher the salinity value, the longer the pneumatophores were. The longest roots came from the rural area (57.6 cm), while the shortest were from the area surrounding the port (34.4 cm) (Figure 1). The pH value was 8.0 and did not differ between locations (data not shown).

### 2.2. Weight of MPs in Soil Samples

**Figure 2:**
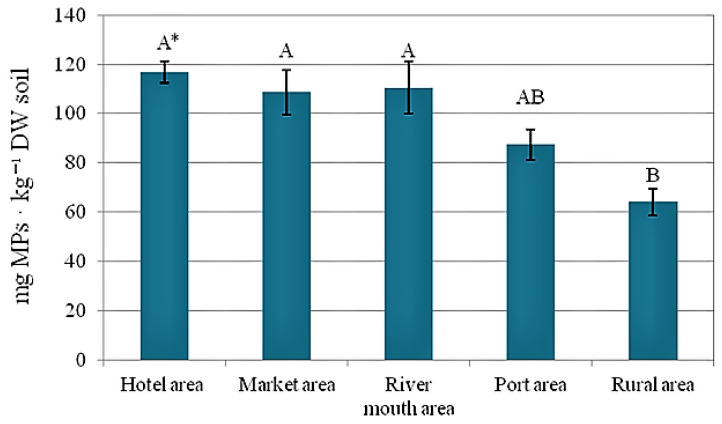
Weight of MPs in the soil from five mangrove areas in Bima City Bay. Data are mean ± SE, *n* = 5. * Capital letters indicate significant differences between weights of MP.

The mean weights of the MPs separated from the soil samples collected in the experimental locations are presented in Figure 2. Samples from the mangrove vegetation near the hotel area revealed the highest MP content (116.9 mg per kg of dry soil). Large amounts of plastic were also found in soil samples from the market and river mouth areas. Significantly the smallest amounts of MPs were found in the soil samples from the rural location, with an average MP mass of 64.2 mg per kg of dry soil (Figure 2).

### 2.3. Discriminant Analysis of Soil and Pneumatophore Samples Based on Presence of Plastic

**Figure 3:**
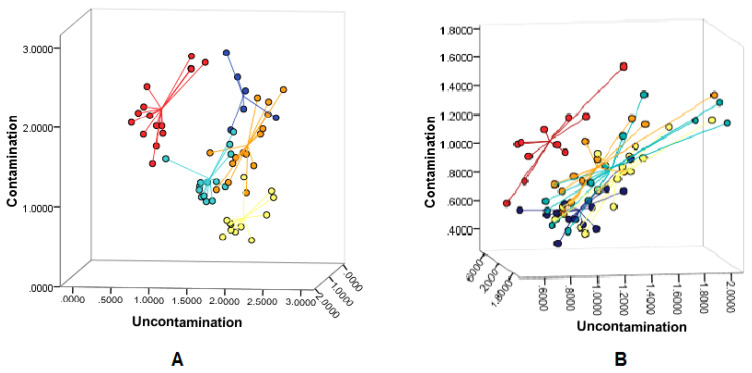
Distances between the centroids of soil samples (**A**) and pneumatophores (**B**); red—rural area; orange—market area; turquoise—port area; dark blue—hotel area; yellow—river mouth; no contamination—apparently very low plastic content in (rural) site (therefore, used as a reference), contamination—four anthropogenized locations visibly polluted with plastic.

The distances between centroid groups indicated that in the rural site, both soil and pneumatophores generated different IR spectra, due to different polymer compositions, compared to the samples taken from the more anthropogenized locations (Figure 3).

Obtained distance values between different centroids are presented in Table 1. For both soil and pneumatophore samples, the distances between samples from the rural area and the samples from the port area were the closest (1.076 and 0.387, respectively) (Table 1). The experimental site with the farthest centroid from the rural location was the river mouth, with 1.738 for the soil and 0.534 for pneumatophores (Table 1).

The soil sample group from the hotel area showed the most similar spectral signals resulting from chemical properties similar to those of samples from the market area; the distance between them was 0.657 (Table 1). High similarity also characterized soil samples from the river mouth and the port area (0.665). Among the soil samples from the anthropogenized zones, the farthest apart were those from the hotel site and the river mouth (1.613) (Table 1).

As for the pneumatophore samples, the distances between centroids of groups from the anthropogenized locations showed that those from the marketplace possessed chemical compositions very close to those of samples from the hotel area, with the distance between them being only 0.071 (Table 1). The farthest distance between centroids, thus the greatest difference in chemical properties, was recorded between groups from the market area and the river mouth (0.378) (Table 1).

### 2.4. Polymer Identification

**Figure 4:**
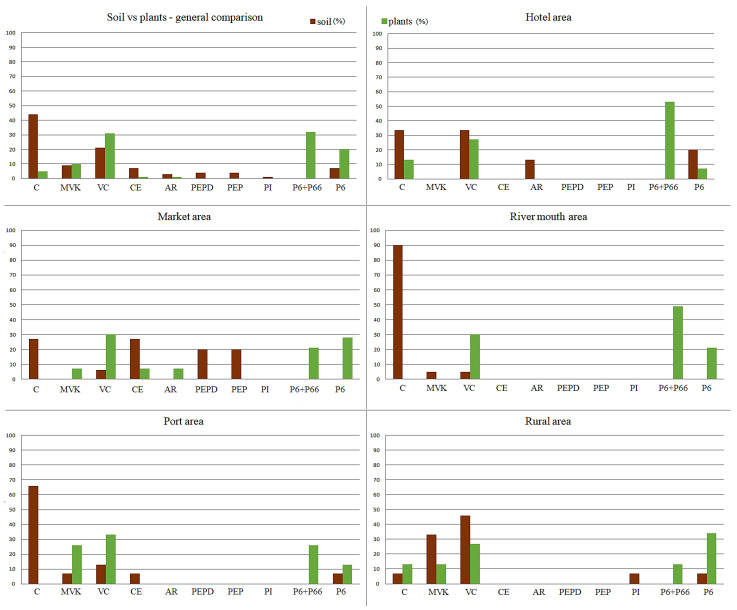
Percentages of polymers identified in the overall spectra of soil and plant (pneumatophore) samples (general comparison), and in the particular experimental areas. Abbreviations: C—cyanoguanidine; MVK—methyl vinyl ketone; VC—vinylidene chloride; CE—cellophane; AR—alkyd resin; PEPD—poly(ethylene: propylene: diene); PEP—poly (ethylene: propylene); PI—poly (isobutene); P6+P66—polyamide 6 + polyamide 66; P6—polyamide 6.

Polymers detected in soil and pneumatophore samples from the experimental sites are shown in Figure 4. In total, nine polymers were identified in the soil from five experimental locations, and seven polymers were found in the pneumatophores. The differences in the soil and pneumatophore polymer composition were related to differences in the percentages of individual polymers. The presence of poly (ethylene: propylene: diene), poly (ethylene: propylene), poly (isobutene) was detected only in the soil, while polyamide 6 and polyamide 66 were associated solely with pneumatophores.

The smallest numbers of polymer types in soil samples were found in those from the river mouth and hotel areas. In most locations, cyanoguanidine was identified, being especially abundant in the river mouth and port areas. The lowest percentage of cyanoguanidine was detected in the rural area. On the other hand, in contrast to the rural site, vinylidene chloride was less common in the polluted areas. Samples from the market and hotel areas showed the most even percentages of detected polymers.

In pneumatophores, the least diverse polymer composition was found in samples from the river mouth, whereas the highest diversity was recorded in the market area. The polymers mostly prevalent in plants were identified as polyamide 6 and polyamide 66 along with vinylidene chloride, especially in mangrove samples from the river mouth and the hotel zone. In the rural samples, polyamides were less numerous.

### 2.5. Trace Element Detection

Recorded concentrations of Mn, Mo, Ni, Pb and Zn were higher in pneumatophores, while concentrations of Ba, Fe, Cd, Co, Cr and Cu were higher in soil sediments (Table 2). Concentrations of all elements were differentiated between the studied areas; however, the differences were not always statistically significant (Table 2).

In the case of soil samples, the hotel area was characterized by the lowest concentrations of Ba and heavy metals, while the river mouth, the market and the port areas were more polluted (Table 2). The river mouth seemed to be the most polluted location, with the highest or mutually highest concentrations of Cd, Co, Cr, Cu, Mo, Ni, Pb and Zn. The rural area revealed higher concentration values than the hotel zone, but lower values than the other sites (Table 2).

The trace elements found in pneumatophores revealed ambiguous patterns (Table 2). Concentrations of Ba and heavy metals, except for Pb, were usually the lowest in samples from the river mouth and port areas. The rural area, in turn, was characterized by significantly higher concentrations of Fe, Ba, Co, Mn and Pb (Table 2).

## 3. Discussion

Plastics and TEs were found in all examined locations in Bima City Bay, both in soil sediments and pneumatophores, but their concentrations/amounts and chemical compositions varied between locations.

### 3.1. Contamination, Chemical Composition and Distribution of MPs in Bima City Bay

Mean amounts of MPs recorded in this work from the sediments were very high, ranging from 64.2 to 117.3 mg·kg^−1^ of soil dry weight. These concentrations were considerably higher than recorded in cultivated regions in China (0 to 0.5 mg·kg^−1^) [52,53] and agricultural and grassland areas in Iran (0.2 to 1.2 mg·kg^−1^) [54], Sweden (0.3 to 3.4 mg·kg^−1^) [55] and Chile (0.6 to 12.9 mg·kg^−1^) [56], but lower than the 915 mg·kg^−1^ found in a municipal area in Germany [57] or the extremely high values obtained from industrial zones in Sydney, Australia, where amounts of MPs in the soil ranged from 300 to 67,500 mg·kg^−1^ (DW) [58]. In Denmark, amounts of MPs found in agricultural soil samples ranged from 0 up to 224.3 mg·kg^−1^ (with a median value of 5.8 mg·kg^−1^ for areas fertilized with sewage sludge and 12 mg·kg^−1^ for untreated soil) [59] and were the most similar to the values obtained in this work. This means that the amounts of MPs in the sediments in Bima City Bay were higher than those recorded on agricultural land, but lower than those of heavily industrialized or urbanized areas. Large amounts of MPs in Bima Bay can be explained by the lack of care for the environment by the inhabitants of the city and bay and possibly inefficient waste management. Microplastic is a very mobile, generally mismanaged substance, which continuously enters the environment as a result of everyday human activities. Darmawan [60] reported that 61.13% of Bima City residents did not discard garbage into government-managed landfills, while 13.97% admitted discarding garbage directly into water areas such as the nearby river and sea. This explains the large amounts of MPs close to the market and the port. The very high amounts of MPs in the vicinity of the hotel area may be a result of pollution drift from the nearby market. MPs were also found in the rural area; although the number of MPs in this location was significantly lower, it could still be considered relatively high. It seems that although MPs have been a problem for a relatively short time, their presence in the environment has become widespread regardless of the degree of anthropogenization of the location.

Microplastics, especially air and water pollutants, can quite easily move through the environment and contaminate areas distant from their emission sources [61]. The migration of MPs in the environment is affected by the type of pollution and the characteristics of the site. This is no different in Bima Bay, where pollution has been found not only in the highly anthropogenized city, but also in a rural location more than 30 km away from urbanized areas. In a rural location that was free from everyday human activity and industry, the most important sources of mangrove pollution, distinct and quite numerous types of polymers were detected. The lack of plastic sources in the rural area suggests their influx into this location. This site is situated at the top of a narrow and elongated bay wherein waves and currents naturally push in and deposit pollutants that are emitted in other areas. Additionally, the mangrove roots are very prone to trapping plastic [43]. The discriminant analysis showed that in the rural area, both the sediments and mangrove pneumatophores were chemically significantly different from those in other locations. This may suggest that some plastics migrate in the environment more easily than others. Substances that distinguished the sediments in the rural area from those at other locations were methyl vinyl ketone and vinylidene chloride. Vinylidene chloride is used to make plastics, including flexible films (e.g., food wrap) and packaging materials. It is also utilized to make flame-retardant coatings for fiber and carpet backings and in piping, coatings for steel pipes, and adhesive applications [62]. It is a dangerous and toxic compound [63]. The analysis of an accidental spill from a damaged tank proved that the substance could migrate through the soil layers within an area of almost 7 ha and reach a lake and wells [64]. Methyl vinyl ketone is a cytotoxic compound classified as industrial waste, as it is utilized in plastics, resins, pesticides, and steroids and in vitamin A and perfume manufacturing. It is also present in tobacco smoke and exhaust gases [65,66]. It has potential for spreading in the environment due to its solubility in water and high volatility [67]. Another location where the chemical composition of sediments was different than in other sites was the river mouth area. Plastic contamination of the river mouth area probably came from different sources than in the port and market areas. Land sources were probably dominant in the river mouth area. Plastic pollution in the oceans usually comes from plastic disposal on land, and unfortunately, it increases every year [68,69]. According to Atwood et al. [70] 70–80% of the MPs on beaches come from activity on land, e.g., due to improper waste management in residential areas [71]. Interestingly, only three types of plastics were identified in the river mouth area, the fewest among all locations studied. The low diversity of polymers in the river mouth area may be due to water currents of the flowing river in which plastic particles are more mobile and deposition in sediment is hindered. The remaining locations (market, port and hotel areas) did not differ significantly in terms of chemical composition. In sediments of mangroves growing near the market, as many as six types of plastics were found. The great variety of plastics around the market area can be explained by the presence of commodities, packages and wrappings of all kinds that are sources of polypropylene, typically used in the production of plastic bags and single-use items, but that were not found in the other locations. Moreover, there are not enough rubbish bins at the Bima market, and the residents still lack ecological awareness that could help limit the littering of water bodies [60]. It is also surprising that there were no significant differences in the chemical composition of samples taken from the hotel area, the port and the market. The reason is probably the migration of polymers from the market to the nearby recreational and hotel areas.

### 3.2. Contamination, Chemical Composition and Distribution of TEs in Bima City Bay

In contrast to plastics, detected concentrations of TEs, particularly heavy metals, in sediments and mangrove pneumatophores were very low regardless of the sampled location and level of anthropogenization. This indicates that the bay, at least the coastal zone, is free from heavy metal pollution. For comparison, soil and *A. alba* pneumatophore samples collected in formerly industrialized Homebush Bay, Australia, were much more contaminated with TEs (Cd: 1.4–3.3 and 0.03–0.57μg·g^−1^, Co: 7.0-20 and 0.56–6.7 μg·g^−1^, Cr: 99–200 and 1.3–23 μg·g^−1^, Cu: 50–156 and 19–49 μg·g^−1^, Mn: 93-460 and 38-450 μg·g^−1^, Ni: 15–48 and 0.97–8.8 μg·g^−1^, Pb: 230–520 and 2.8–98 μg·g^−1^, and Zn: 254–680 and 24–193 μg·g^−1^ in soil and plants, respectively) [72]. High concentrations of Cu, Pb, Mn, Fe, and Zn were recorded in A. officinalis leaves in Tamil Nadu, India [73] and in *A. marina* roots in the Persian Gulf [74] and highly saline Red Sea coast [46]. Low concentrations of TEs in sediments and mangrove organs are also not unusual. A low accumulation (i.e., below the maximum residue levels) of heavy metals in mangrove leaves was found in the natural area of Tuticorin coast (Hare Island), Gulf of Mannar Marine Biosphere Reserve, India [75]. An explanation for the lack of TE contamination could be the general characteristics of Bima City Bay. It is a site moderately urbanized without intensively industrialized zones. For this reason, the soil sediment was not contaminated with TEs, and consequently, the concentrations of heavy metals were very low in the pneumatophores of *A. alba*. It is worth noting that, even if the concentrations of TEs in the sediments were higher, a high pH would favor the precipitation of metals rather than their uptake by plants. Locations with the greatest anthropopressure (the market area, port area and especially river mouth area) were characterized by sediments more contaminated with TEs. Higher sediment contamination with trace elements in urban and commercial areas was also found in the Potomac and Anacostia rivers [76]. The highest concentrations of TEs in sediments were recorded in the river mouth area, where water currents systematically deposit element-rich sediments amassed along the river’s course. The lowest concentrations of heavy metals in the soil around the hotels could be explained by the fact that tourist zones must be well-maintained to remain attractive, thus no toxic pollution would have been allowed. Somewhat surprising findings were the presence of TEs in the rural area at the same levels as those in highly anthropogenic locations, and the significantly higher concentrations of TEs in mangrove pneumatophores growing in this location. This might be linked to the above assumption, namely that due to its position, this location is affected by pollutants inflowing from Bima city. Moreover, 6 km from the sampling site, there is an airport. It should be noted, however, that despite significant differences, the concentrations of TEs in the pneumatophores of the mangrove trees were very low in all locations and did not pose a threat to plants.

### 3.3. Composition of Plastics and Concentrations of TEs in the Soil and Plant Samples

A comparison between the composition and percentages of sediment-associated MPs and plastics detected in pneumatophores (the source of which could probably be NPs trapped in the sediments or suspended in the water or air) indicated the varying affinity of plant tissues for polymer types, resulting in selective absorption or adsorption on the pneumatophores’ surfaces. The selectivity of absorption/adsorption could also be inferred from the Mahalanobis distances obtained from the discriminant analysis of plant- and soil-associated plastics. Values calculated for plant samples were lower than to those for the soil samples. This indicated more complex polymer composition in the sediments. Sediments sampled from the market area, one of the most polluted locations, contained poly (ethylene: propylene), poly (ethylene: propylene: diene), cellophane, vinylidene chloride and cyanoguanidine, while the plastic content in pneumatophores was similar only regarding the presence of vinylidene chloride and cellophane. Similarly, in the river mouth, despite only identifying three types of plastic, selectivity in their absorption/adsorption by plants was noticeable. In the sediments, cyanoguanidine, vinylidene chloride and methyl vinyl ketone were found. Of these, only the vinylidene chloride was also present in the pneumatophore samples.

An interesting case is cyanoguanidine. This polymer was detected in the sediment of each experimental site and was particularly abundant in the river mouth and the neighboring port. The presence of cyanoguanidine in sediments did not correspond with its accumulation in the pneumatophores. Plant samples revealed zero to low percentages of this nitrile. The two types of polypropylene, which could be detected solely in the soil neighboring the market, were not observed in the pneumatophores of *A. alba*. Additionally, the polyisobutene that distinguished the soil from the rural location was not found in the mangroves at all. The reverse and at the same time clearest trend could be seen in polyamides. In each location, their content in plant pneumatophores was considerably higher than in the soil. Vinylidene chloride also showed a tendency to accumulate in pneumatophores, regardless of its abundance in the soil. The alkyd resin was mostly absent in the soil and pneumatophores or occurred at low levels, whereas the cellophane and methyl vinyl ketone revealed the most ambiguous patterns of soil and tissue concentrations. It is also worth noting that the chemical differences shown in the discriminant analysis were smaller in pneumatophore samples than in soil samples. This may suggest that only selected polymers can be taken up from the sediments and accumulated by pneumatophores or adsorbed on their surfaces. There are no studies on absorption/adsorption of plastics by mangrove pneumatophores so far. It is noteworthy, however, that due to evolutionary adaptations to a harsh environment, it is not easy for mangrove roots to absorb unknown chemical particles from the environment [77]. Lignification of the exodermis could directly prevent excessive amounts of chemical compounds from entering the roots [78]. This could, at least to some extent, explain why the plastic composition detected in the mangrove spectra was less diverse (lower distances between centroids) than in the soil spectra.

Several reviews have demonstrated that plants other than mangroves can adsorb and absorb nanoplastics [79,80,81]; however, no detailed data are available on selective accumulation when a variety of substances are present in the soil. Research performed on the model of *Arabidopsis thaliana* revealed that the accumulation of polystyrene-based nanoplastics and its influence on plants depends on the particles’ charge [82]. Plastic absorption and accumulation may cause adverse effects on plants, as it affects the environment in which plants grow. It happens through changes in the physico-chemical properties of the soil by causing alterations in the water cycle and soil–plant interactions [83,84]. Contamination with MPs harmfully impacts the microbial communities in the environment, as biofilms covering MP particles and natural substances differ considerably in their composition of microorganisms [17]. Finally, plastics may interact with cell membranes and organelles, causing direct toxicity and physical injuries [79,85]. Pignattelli et al. [13] described acute and chronic toxicity caused by polymers, especially PVC, in *Lepidium sativum*. Additionally, as elaborated herein, plastic accumulation in the environment could have a negative effect on *A. alba* plants. The lengths of pneumatophores of *A. alba* plants were the shortest in the hotel area, the location with the highest concentrations of MPs in its sediments.

In general, Bima Bay is not polluted with TEs. Concentrations of TEs both in sediments and plants were very low. Slightly higher concentrations of Ba, Fe, Cd, Co, Cr and Cu were recorded in sediments, and of Mn, Mo, Ni, Pb and Zn in mangrove roots. A particularly interesting result was obtained for Pb. Despite the high pH (slightly alkaline) and low mobility of Pb in the environment, in every experimental location its concentration in pneumatophores was higher than in the soil. This is in contrast to observations made in Australia, on *A. marina* pneumatophores, in which the amount of Pb was always lower than in soil [72]. In addition, research carried out on mangrove vegetation from Ennore (east coast of India) pointed out that more Pb was detected in sediments than in *A. marina* roots [86]. The accumulation of Pb by mangroves growing in Bima Bay was certainly extended in time and not necessarily related to the current level of Pb in the sediments.

MPs may be TE vectors in the environment. Trace element sorption onto MPs exhibits a clear dependence on pH—with increasing pH in water, adsorption of TEs on MPs increases [28,76]. However, as pH continues to increase to neutral or alkaline levels, TEs may not be as readily adsorbed due to passivation or precipitation [28]. In the locations in this study with the highest TE concentrations (river mouth, port and market areas), cyanoguanidine was also abundant, which may suggest that this polymer has a considerable affinity for TEs, despite the high pH.

## 4. Materials and Methods

### 4.1. Experimental Locations

**Figure 5:**
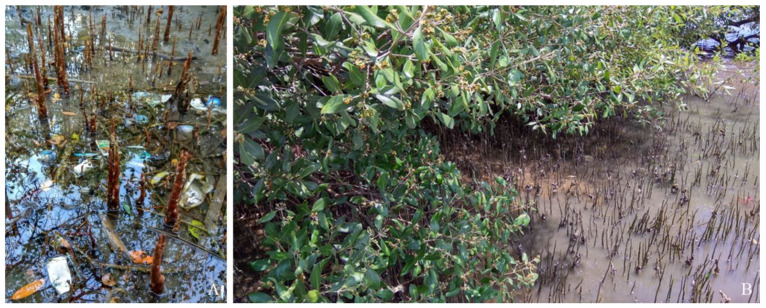
Sampling locations in market (**A**) and rural areas (**B**).

**Figure 6:**
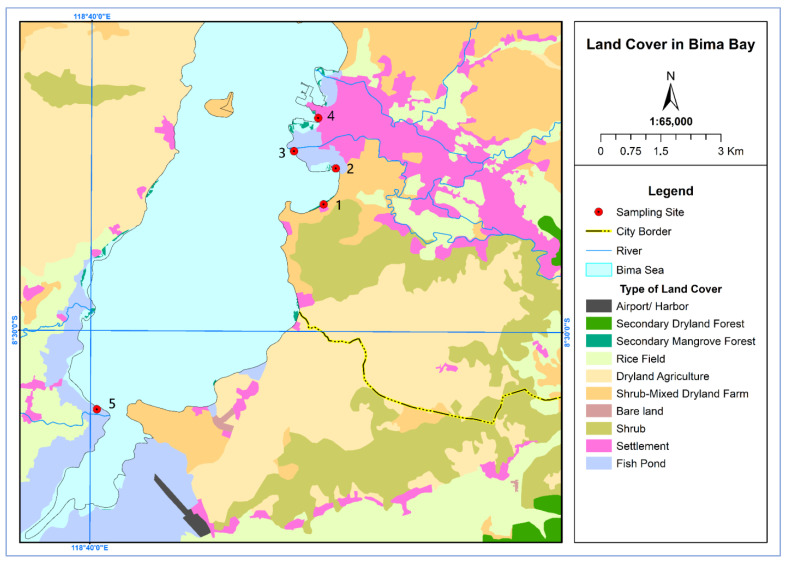
Map of the five sampling locations in Bima City Bay.

Experimental locations were selected in five mangrove areas in the bay of Bima City, characterized by different degrees of anthropopressure. Bima is the largest and most populated city on Sumbawa island. Four areas showed contamination with MPs (visible presence of plastic) and included mangrove thickets neighboring a hotel area (Spot 1, 2.38 hectares, Figure 5A), market area (Spot 2, 0.38 hectares), river mouth area (Spot 3, 0.37 hectares) and port area (Spot 4, 0.41 hectares). The last and most remote area, which seemed to be free of plastic pollution (no apparent presence of plastics) was the rural area (Spot 5, 4.72 hectares, Figure 5B). The experimental areas are shown in Figure 6.

Details of human activity and contamination degrees in the experimental locations are presented in supplementary materials (Table S1).

In every experimental location, the pH value (pH stripes, Macherey Nagel) and salt concentration (refractometer TI-SAT100, Trans Instruments) in the water were measured using field methods.

### 4.2. Sample Collection

Soil (eutric regosol) and pneumatophore samples were collected pairwise from each experimental location, in five replicates (sub-locations) during low tide. The sub-location spots within each location were determined by the point intercept transect (PIT) method; the distance between sub-locations was 25 m. The total transect length at each location was 100 m.

Soil samples were collected by inserting a 7.62 cm diameter PVC pipe into the soil to 10 cm depth. Collected soil samples were dried in an oven at 90 °C for 24 h and stored in labeled plastic bags.

Pneumatophore samples were taken from the mangrove species *Avicennia alba* Blume, 1826 (Lamiales: Acanthaceae), which is categorized as a tree and is the dominant mangrove species in Bima City. The soil around each pneumatophore was dug using a scoop until the cable root was clearly seen. The samples were removed from the root by knife and cleaned with running tap water. Gentle hand washing (c. 1 min) was performed in order to remove mud. Sampled pneumatophore parts started from the root tip, and were harvested together with the whole part located above the ground surface. Collected samples were dried as described above. The length of the dry fragment was measured with a ruler.

### 4.3. Quantitative Assessment of the Presence of Microplastics in Soil Sediments

The modified sediment microplastic isolation (SMI) method was used to separate MPs from the soil sediments [87]. Dry soil (50 g) was mixed with NaCl solution (1.2 g cm^−3^, 700 mL). The obtained solution was stirred for 5 min in the SMI unit with a magnetic stirrer and then was left for 5 min. until the sediment settled to the bottom. When the supernatant was clear, the valve was closed, and the supernatant in the head chamber was filtered with a 30 mm diameter nylon mesh. Then, MPs were separated from macroplastics according to size. Fragments larger than 5 mm were excluded from the analyses. Collected MPs were placed on Petri dishes and weighed per sub-location and presented as mg of MPs per kg of dry weight (DW) soil.

### 4.4. Analysis of MPs with Fourier Transform Infrared Spectroscopy (FT-IR)

Plastics contained in both soil and the pneumatophore tissues of mangroves growing in all specified locations was analyzed with FT-IR spectroscopy, allowing the study of chemical composition based on specific chemical bonds of polymers [18]. For this purpose, 1 mg of MP, extracted from the soil samples or powdered mangrove pneumatophores, and 300 mg of high-grade purity potassium bromide powder (KBr) were transferred into an agate mortar and ground together with an agate pestle until both were well mixed and had the consistency of fine powder. The obtained mixture was pressed into pellets with the use of a dedicated pellet-maker device and a hydraulic press with a pressure of 10–15 tons. The pellets were then placed into a pellet-holder, which was in turn placed in the measuring chamber of a Perkin-Elmer System 2000 spectrometer.

Pegram’s 2000 software operating on a Windows system managed the measurement procedure. The spectrum of each sample was registered in the medium IR spectral range, i.e., 4000–400 cm^−1^. The resolution was set to 1 cm^−1^. Twenty-five scans were performed for both background spectrum (no sample in the measuring chamber) and the working sample.

### 4.5. Polymer Identification

**Figure 7:**
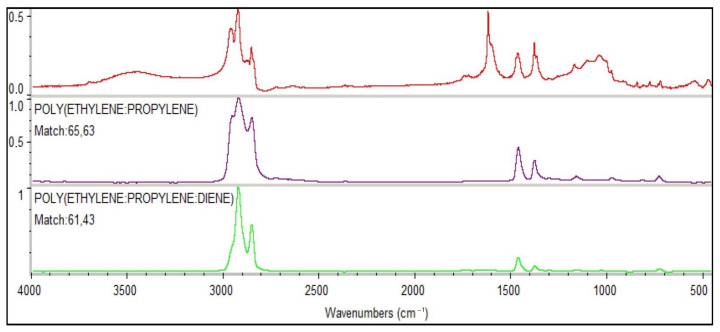
The appearance of the polymer type with the highest compatibility rating with the reference spectra displayed by the OMNIC 9 software.

Thermo Fisher Scientific’s OMNIC 9 software was applied for the identification of polymers present in the soil and pneumatophore fragments. Experimentally registered spectra of each sample were entered into a software database and matched with the reference spectra (Hummel Polymer Sample Library and the HR Nicolet Sampler Library). Because soil and pneumatophore samples were not previously purified with the wet peroxide oxidation (WPO) method (which could have damaged the samples), the compatibility of polymers was inferred from the compound rating displayed by the software, not from the percentage value (Figure 7).

### 4.6. Plant Metal Extractions and ICP-OES

All dried pneumatophores were individually hammer-milled (Retsch SM100) to obtain a fine powder. To determine total trace metal concentrations in the biomass, this powder was wet-digested in Pyrex glass tubes in a heating block. The digestion consisted of three cycles in 1 mL HNO_3_ (70%) and one cycle in 1 mL HCl (37%) at 120 °C for 4 h. Samples were thereafter dissolved in HCl (37%) and diluted to a final volume of 5 mL (2% HCl) with Millipore water. The extracts were subsequently analyzed with inductively coupled plasma optical emission spectrometry (ICP-OES, Agilent Technologies 700 Series). All samples (five biological replications per location) were examined at least in triplicate. Blanks and certified reference material R (trace elements in spinach, Standard Reference Material 1570a, National Institute of Standards and Technology, USA, Department of Commerce) were included for quality control of the data. The recovery percentages of Cd, Cu, Fe, Mn, Mo, Ni and Zn varied from 72.0% (Cu) to 92.2% (Fe, Zn).

### 4.7. Soil Metal Extractions and ICP-OES

Soil was oven-dried (60 °C) and sieved (<2 mm). Pseudo-total metal concentrations of the soil samples were estimated by *aqua regia* digestion [88]. For this, 0.5 g of oven-dried soil was microwaved (MLS 1200 Mega high performance microwave digestion, Milestone, Italy) and digested in an HNO_3_-HCl solution (1:3 *v*:*v*) at 160 °C (25 min ramp time, 10 min ventilation). The extracts were analyzed using the ICP-OES as described above. A reference soil (CRM 143 R Sewage Sludge Amended Soil, Community Bureau of Reference—BCR N 230) was included for confirmation of the analysis. The recovery percentages of Cd, Co, Cr, Cu, Mn, Ni, Pb and Zn varied from 72.3% (Ni) to 105.7% (Co).

### 4.8. Statistical Analysis

SPSS 16 software was used for the statistical evaluation of the length of pneumatophores, level of salinity and weight of microplastics in soil samples. Data were analyzed with ANOVA. The results were subjected to an analysis of variance at a significance level of *p* ≤ 0.05. Tukey’s honestly significant difference (HSD) test was used to determine the significance of differences between the means.

Discriminant analysis with TQ Analyst software (Thermo Fisher Scientific Inc., Waltham, MA, USA) was performed to classify the MP samples based on their spectral differences. The spectral regions were determined by the degree of separability from the sample groups. IR spectra of samples collected in different locations were considered standards. The procedure that differentiates spectra and gathers them into homologous groups is known as discriminant analysis. This is an option in Omnic software used for this type of analysis. It is based on calculation of Mahalanobis distances. They allow grouping samples in homologous groups and separating each group from another if the difference is statistically significant. First, the calculation of the average spectrum out of all spectra used in the calibration process is conducted. Then, a specific algorithm is used to determine distance from the average.

With following notation used: D—distance (scalar); A—a data vector (n × 1); Aaverage—the average data vector (n × 1); M—covariance matrix (n × n); n—the number of data points in A, (A—A average)—the transpose of (A—A Aaverage), the formula:

$$\sqrt{{\left(A-Aaverage\right)}^{T}\times M^{-1}\times \left(A-Aaverage\right)\;}$$

is applied to determine the distance of a given sample from the average. This formula is included in the software applied.

Repeated testing allowed for obtaining the correct spectral regions and made it easy for classification and interpretation. Besides testing the samples with the standard procedure, selections of spectral regions were analyzed with the data on the potential wavelengths detecting plastic obtained from the literature. Here, the discriminant analysis was conducted on FTIR spectra with four wavelength ranges: 1700–1500 cm^−1^, 1400–1300 cm^−1^, 1200–1000 cm^−1^ and 900–700 cm^−1^.

The Mahalanobis distances obtained from the discriminant analysis of MP samples were used to find the centroid points of each sample group. The centroid distances between groups indicated similarities/differences in chemical composition of each sample group [89]. The samples used to calculate a given centroid were similar to each other at a statistically significant level, in terms of chemical bonds present in them; hence, principal components obtained from spectral data registered for each sample formed a homologous group (centroid). If the sample was out of a given centroid (i.e., belonged to another centroid-homologous group) it meant that this sample was different (had different chemical bonds) from those forming the given centroid.

## 5. Conclusions

The results suggest that microplastics, rather than trace elements, are the main pollution issue in Bima City Bay sediments. The accumulation of different plastics in the environment may result in their adsorption/absorption into air root tissues of *A. alba*. The detection of plastics with slightly different compositions in the roots compared to those in soil sediments indicated a differential affinity of polymers for pneumatophores.

## Figures and Tables

**Table 1:** Distance values between centroids of soil and pneumatophore samples from five mangrove areas in Bima City Bay.

Location	Soil					Pneumatophores				
	Rural Area	Hotel Area	Market Area	River Mouth Area	Port Area	Rural Area	Hotel Area	Market Area	River Mouth Area	Port Area
Rural area	0					0				
Hotel area	1.146	0				0.524	0			
Market area	1.215	0.657	0			0.471	0.071	0		
River mouth area	1.738	1.613	0.958	0		0.534	0.345	0.378	0	
Port area	1.076	1.231	0.702	0.665	0	0.387	0.137	0.095	0.329	0

**Table 2:** Concentrations (mg·kg^−1^) of TEs found in sediments and pneumatophores of *A. alba*. Data are mean ± SE, *n* = 5.

Location	Fe	Ba	Cd	Co	Cr	Cu	Mn	Mo	Ni	Pb	Zn
	Soil										
Rural area	3099 ^b^* (±59.5)	7.09 ^b^ (±0.73)	0.25 ^b^ (±0.01)	1.65 ^b^ (±0.02)	1.27 ^bc^ (±0.04)	3.60 ^b^ (±0.52)	90.5 ^a^ (±18.5)	0.11 ^b^ (±0.01)	0.67 ^b^ (±0.04)	0.80 ^bc^ (±0.09)	7.55 ^b^ (±0.42)
Hotel area	2015 ^c^ (±104)	5.69 ^b^ (±0.02)	0.14 ^c^ (±0.01)	1.17 ^c^ (±0.04)	1.09 ^c^ (±0.06)	1.06 ^c^ (±0.16)	46.1 ^b^ (±2.84)	0.19 ^ab^ (±0.01)	0.43 ^c^ (±0.04)	0.42 ^c^ (±0.06)	3.51 ^c^ (±0.41)
Market area	3459 ^ab^ (±91.5)	17.4 ^a^ (±3.08)	0.30 ^ab^ (±0.01)	2.00 ^a^ (±0.08)	1.31 ^bc^ (±0.04)	4.05 ^ab^ (±0.41)	66.3 ^ab^ (±7.40)	0.23 ^a^ (±0.02)	0.83 ^b^ (±0.04)	1.69 ^ab^ (±0.36)	8.39 ^b^ (±0.57)
River mouth area	3684 ^a^ (±122)	12.0 ^ab^ (±0.46)	0.35 ^a^ (±0.02)	2.04 ^a^ (±0.07)	1.79 ^a^ (±0.10)	5.53 ^a^ (±0.32)	72.5 ^ab^ (±5.89)	0.22 ^a^ (±0.03)	1.04 ^a^ (±0.07)	1.88 ^a^ (±0.30)	13.7 ^a^ (±1.45)
Port area	3762 ^a^ (±155)	10.2 ^b^ (±1.59)	0.34 ^a^ (±0.03)	2.19 ^a^ (±0.09)	1.36 ^b^ (±0.05)	2.65 ^bc^ (±0.49)	76.0 ^ab^ (±7.31)	0.18 ^ab^ (±0.03)	0.83 ^b^ (±0.05)	0.83 ^bc^ (±0.08)	7.91 ^b^ (±0.46)
	Pneumatophores										
Rural area	1130 ^a^ (±162)	3.57 ^a^ (±0.32)	0.05 ^b^ (±0.01)	0.54 ^a^ (±0.06)	1.30 ^a^ (±0.18)	1.98 ^bc^ (±0.28)	126 ^a^ (±26.6)	0.23 ^a^ (±0.05)	1.20 ^a^ (±0.19)	6.08 ^a^ (±0.41)	14.0 ^a^ (±1.11)
Hotel area	738 ^ab^ (±133)	1.45 ^b^ (±0.21)	0.08 ^a^ (±0.00)	0.26 ^b^ (±0.02)	1.12 ^a^ (±0.27)	1.39 ^c^ (±0.13)	34.1 ^b^ (±3.56)	0.27 ^a^ (±0.05)	2.42 ^a^ (±0.95)	5.09 ^ab^ (±0.39)	11.5 ^a^ (±1.44)
Market area	724 ^ab^ (±130)	3.14 ^a^ (±0.44)	0.06 ^ab^ (±0.01)	0.39 ^ab^ (±0.05)	0.74 ^a^ (±0.15)	3.49 ^a^ (±0.35)	41.0 ^b^ (±4.45)	0.16 ^a^ (±0.01)	0.82 ^a^ (±0.05)	4.04 ^b^ (±0.08)	11.1 ^a^ (±0.98)
River mouth area	471 ^b^ (±41.3)	1.62 ^b^ (±0.14)	0.04 ^bc^ (±0.00)	0.34 ^b^ (±0.03)	1.63 ^a^ (±0.80)	2.56 ^ab^ (±0.11)	36.8 ^b^ (±3.46)	0.25 ^a^ (±0.07)	2.89 ^a^ (±1.48)	3.67 ^b^ (±0.41)	11.3 ^a^ (±1.83)
Port area	533 ^b^ (±61.9)	2.37 ^ab^ (±0.23)	0.03 ^c^ (±0.00)	0.31 ^b^ (±0.02)	0.83 ^a^ (±0.03)	1.61 ^bc^ (±0.21)	34.0 ^b^ (±4.58)	0.13 ^a^ (±0.02)	1.13 ^a^ (±0.31)	5.62 ^a^ (±0.42)	11.4 ^a^ (±1.08)

* Different letters indicate significant differences between locations.

## Data Availability

Not applicable.

## Supplementary Materials

The following supporting information can be downloaded at: https://www.mdpi.com/article/10.3390/plants12030462/s1, Table S1: Experimental areas.

## References

[1] Hildering A., Keessen A., van Rijswick H.F. (2009). Tackling pollution of the Mediterranean Sea from land-based sources by an integrated ecosystem approach and the use of the combined international and European legal regimes. Utrecht Law Rev..

[2] Tiquio M.G.J.P., Marmier N., Francour P. (2017). Management frameworks for coastal and marine pollution in the European and South East Asian regions. Ocean Coast. Manag..

[3] Bonanno G., Veneziano V., Orlando-Bonaca M. (2020). Comparative assessment of trace element accumulation and biomonitoring in seaweed *Ulva lactuca* and seagrass *Posidonia oceanica*. Sci. Total Environ..

[4] Arisekar U., Shakila R.J., Shalini R., Jeyasekaran G. (2021). Pesticides contamination in the Thamirabarani, a perennial river in peninsular India: The first report on ecotoxicological and human health risk assessment. Chemosphere.

[5] Adyasari D., Pratama M.A., Teguh N.A., Sabdaningsih A., Kusumaningtyas M.A., Dimova N. (2021). Anthropogenic impact on Indonesian coastal water and ecosystems: Current status and future opportunities. Mar. Pollut. Bull..

[6] Hodson M.E., Duffus-Hodson C.A., Clark A., Prendergast-Miller M., Thorpe K.L. (2017). Plastic Bag Derived-Microplastics as a Vector for Metal Exposure in Terrestrial Invertebrates. Environ. Sci. Technol..

[7] Willis K.A., Eriksen R., Wilcox C., Hardesty B.D. (2017). Microplastic Distribution at Different Sediment Depths in an Urban Estuary. Front. Mar. Sci..

[8] Wicaksono E.A., Werorilangi S., Galloway T.S., Tahir A. (2021). Distribution and seasonal variation of microplastics in Tallo river, Makassar, Eastern Indonesia. Toxics.

[9] Dusaucy J., Gateuille D., Perrette Y., Naffrechoux E. (2021). Microplastic pollution of worldwide lakes. Environ. Pollut..

[10] Barros J., Seena S. (2021). Plastisphere in freshwaters: An emerging concern. Environ. Pollut..

[11] Pehlivan N., Gedik K. (2021). Particle size-dependent biomolecular footprints of interactive microplastics in maize. Environ. Pollut..

[12] Wright S.L., Kelly F.J. (2017). Plastic and human health: A micro issue?. Environ. Sci. Technol..

[13] Pignattelli S., Broccoli A., Renzi M. (2020). Physiological responses of garden cress (*L. sativum*) to different types of microplastics. Sci. Total Environ..

[14] Frias J.P., Nash R. (2019). Microplastics: Finding a consensus on the definition. Mar. Pollut. Bull..

[15] Rochman C.M., Hoh E., Kurobe T., Teh S.J. (2013). Ingested plastic transfers hazardous chemicals to fish and induces hepatic stress. Sci. Rep..

[16] Prajapati A., Narayan Vaidya A., Kumar A.R. (2022). Microplastic properties and their interaction with hydrophobic organic contaminants: A review. Environ. Sci. Pollut. Res..

[17] Cholewińska P., Moniuszko H., Wojnarowski K., Pokorny P., Szeligowska N., Dobicki W., Polechoński R., Górniak W. (2022). The Occurrence of Microplastics and the Formation of Biofilms by Pathogenic and Opportunistic Bacteria as Threats in Aquaculture. Int. J. Environ. Res. Public Health.

[18] Ahmed M.B., Rahman M.S., Alom J., Hasan M.S., Johir M.A.H., Mondal M.I.H., Lee D.Y., Park J., Zhou J.L., Yoon M.H. (2021). Microplastic particles in the aquatic environment: A systematic review. Sci. Total Environ..

[19] Yin K., Wang D., Zhao H., Wang Y., Guo M., Liu Y., Li B., Xing M. (2021). Microplastics pollution and risk assessment in water bodies of two nature reserves in Jilin Province: Correlation analysis with the degree of human activity. Sci. Total. Environ..

[20] Mbengue S., Alleman L.Y., Flament P. (2017). Metal-bearing fine particle sources in a coastal industrialized environment. Atmos. Res..

[21] Duman F., Cicek M., Sezen G. (2007). Seasonal changes of metal accumulation and distribution in common club rush (*Schoenoplectus lacustris*) and common reed (*Phragmites australis*). Ecotoxicology.

[22] Ray R., Mandal S.K., González A.G., Pokrovsky O.S., Jana T.K. (2021). Storage and recycling of major and trace element in mangroves. Sci. Total Environ..

[23] Wattigney W.A., Irvin-Barnwell E., Li Z., Ragin-Wilson A. (2019). Biomonitoring of mercury and persistent organic pollutants in Michigan urban anglers and association with fish consumption. Int. J. Hyg. Environ. Health.

[24] Abdelhady A.A., Khalil M.M., Ismail E., Mohamed R.S., Ali A., Snousy M.G., Fan J., Zhang S., Yanhong L., Xiao J. (2019). Potential biodiversity threats associated with the metal pollution in the Nile–Delta ecosystem (Manzala lagoon, Egypt). Ecol. Indic..

[25] Cao Y., Zhao M., Ma X., Song Y., Zuo S., Li H., Deng W. (2021). A critical review on the interactions of microplastics with heavy metals: Mechanism and their combined effect on organisms and humans. Sci. Total Environ..

[26] Arifin Z., Puspitasari R., Miyazaki N. (2012). Heavy metal contamination in Indonesian coastal marine ecosystems: A historical perspective. Coast. Mar. Sci..

[27] Kusnadi E.A., Triandiza T. (2020). Assessment of sediment quality in the waters around of Ternate city, North of Maluku, Indonesia based on an index analysis approach. IOP Conf. Series Earth Environ. Sci..

[28] Nagajyoti P.C., Lee K.D., Sreekanth T.V.M. (2010). Heavy metals, occurrence and toxicity for plants: A review. Environ. Chem. Lett..

[29] Kabata-Pendias A., Pendias H. (1999). Biogeochemia Pierwiastków Śladowych.

[30] Maghsodian Z., Sanati A.M., Ramavandi B., Ghasemi A., Sorial G.A. (2021). Microplastics accumulation in sediments and *Periophthalmus waltoni* fish, mangrove forests in southern Iran. Chemosphere.

[31] Alongi D.M. (2014). Carbon cycling and storage in mangrove forests. Annu. Rev. Mar. Sci..

[32] Tomlinson P.B. (2016). The Botany of Mangroves.

[33] Usman A.R., Alkredaa R.S., Al-Wabel M.I. (2013). Heavy metal contamination in sediments and mangroves from the coast of Red Sea: *Avicennia marina* as potential metal bioaccumulator. Ecotoxicol. Environ. Saf..

[34] Barbier E.B. (2016). The protective service of mangrove ecosystems: A review of valuation methods. Mar. Pollut. Bull..

[35] Barbier E.B., Hacker S.D., Kennedy C., Koch E.W., Stier A.C., Silliman B.R. (2011). The value of estuarine and coastal ecosystem services. Ecol. Monogr..

[36] Sidik F., Supriyanto B., Krisnawati H., Muttaqin M.Z. (2018). Mangrove conservation for climate change mitigation in Indonesia. Wiley Interdiscip. Rev. Clim. Change.

[37] Santini N.S., Reef R., Lockington D.A., Lovelock C.E. (2015). The use of fresh and saline water sources by the mangrove *Avicennia marina*. Hydrobiologia.

[38] Purnobasuki H. (2011). Structure of Lenticels on the Pneumatophores of *Avicennia marina*: As Aerating Device Deliver Oxygen in Mangrove’s root. J. BIOTA.

[39] Rahaman S., Biswas S.K., Rahaman M.S., Ghosh A.K., Sarder L., Siraj S.M.S., Islam S.S. (2014). Seasonal nutrient distribution in the Rupsha-Passur tidal river system of the Sundarbans mangrove forest, Bangladesh. Ecol. Process..

[40] Zamprogno G.C., Caniçali F.B., dos Reis Cozer C., Otegui M.B.P., Graceli J.B., da Costa M.B. (2021). Spatial distribution of microplastics in the superficial sediment of a mangrove in Southeast Brazil: A comparison between fringe and basin. Sci. Total Environ..

[41] Maghsodian Z., Sanati A.M., Tahmasebi S., Shahriari M.H., Ramavandi B. (2022). Study of microplastics pollution in sediments and organisms in mangrove forests: A review. Environ. Res..

[42] Zhou Q., Tu C., Fu C., Li Y., Zhang H., Xiong K., Zhao X., Li L., Waniek J.J., Luo Y. (2020). Characteristics and distribution of microplastics in the coastal mangrove sediments of China. Sci. Total Environ..

[43] Luo Y.Y., Not C., Cannicci S. (2021). Mangroves as unique but understudied traps for anthropogenic marine debris: A review of present information and the way forward. Environ. Pollut..

[44] Rizal A., Sahidin A., Herawati H. (2018). Economic value estimation of mangrove ecosystems in Indonesia. Biodivers. Int. J..

[45] Garcés-Ordóñez O., Castillo-Olaya V.A., Granados-Briceño A.F., García L.M.B., Díaz L.F.E. (2019). Marine litter and microplastic pollution on mangrove soils of the Ciénaga Grande de Santa Marta, Colombian Caribbean. Mar. Pollut. Bull..

[46] El Ashmawy A.A., Masoud M.S., Yoshimura C., Dilini K., Abdel-Halim A.M. (2021). Accumulation of heavy metals by Avicennia marina in the highly saline Red Sea coast. Environ. Sci. Pollut. Res..

[47] Brennecke D., Duarte B., Paiva F., Caçador I., Canning-Clode J. (2016). Microplastics as vector for heavy metal contamination from the marine environment. Estuar. Coast. Shelf Sci..

[48] Naqash N., Prakash S., Kapoor D., Singh R. (2020). Interaction of freshwater microplastics with biota and heavy metals: A review. Environ. Chem. Lett..

[49] Fu L., Li J., Wang G., Luan Y., Dai W. (2021). Adsorption behavior of organic pollutants on microplastics. Ecotoxicol. Environ. Saf..

[50] Abbasi S., Moore F., Keshavarzi B., Hopke P.K., Naidu R., Rahman M.M., Oleszczuk P., Karimi J. (2020). PET-microplastics as a vector for heavy metals in a simulated plant rhizosphere zone. Sci. Total Environ..

[51] Bradney L., Wijesekara H., Palansooriya K.N., Obadamudalige N., Bolan N.S., Ok Y.S., Rinklebe J., Kim K.H., Kirkham M.B. (2019). Particulate plastics as a vector for toxic trace-element uptake by aquatic and terrestrial organisms and human health risk. Environ. Int..

[52] Zhang G.S., Liu Y.F. (2018). The distribution of microplastics in soil aggregate fractions in southwestern China. Sci. Total Environ..

[53] Zhang S., Liu X., Hao X., Wang J., Zhang Y. (2020). Distribution of low-density microplastics in the mollisol farmlands of northeast China. Sci. Total Environ..

[54] Rezaei M., Riksen M.J., Sirjani E., Sameni A., Geissen V. (2019). Wind erosion as a driver for transport of light density microplastic. Sci. Total Environ..

[55] Ljung E., Olesen K.B., Andersson P.G., Fältström E., Vollertsen J., Wittgren H.B., Hagman M. (2018). Mikroplaster i kretsloppet. Sven. Vatten Utveckl. Rapp..

[56] Corradini F., Meza P., Eguiluz R., Casado F., Huerta-Lwanga E., Geissen V. (2019). Evidence of microplastic accumulation in agricultural soils from sewage sludge disposal. Sci. Total Environ..

[57] Dierkes G., Lauschke T., Becher S., Schumacher H., Földi C., Ternes T. (2019). Quantification of microplastics in environmental samples via pressurized liquid extraction and pyrolysis-gas chromatography. Anal. Bioanal. Chem..

[58] Fuller S., Gautam A. (2016). A procedure for measuring microplastics using pressurized fluid extraction. Environ. Sci. Technol..

[59] Vollertsen J., Hansen A.A. (2017). Microplastic in Danish Wastewater: Sources, Occurrences and Fate.

[60] Darmawan A. (2014). Perilaku Masyarakat dalam Mengelola Sampah di Kota Bima Nusa Tenggara Barat. J. Pembang. Wil. Kota.

[61] Isobe A., Kubo K., Tamura Y., Nakashima E., Fujii N. (2014). Selective transport of microplastics and mesoplastics by drifting in coastal waters. Mar. Pollut. Bull..

[62] National Center for Biotechnology Information PubChem Compound Summary for CID 6366, Vinylidene Chloride. https://pubchem.ncbi.nlm.nih.gov/compound/Vinylidene-chloride.

[63] Short R.D., Winston J.M., Minor J.L., Hong C.B., Seifter J., Lee C.C. (1977). Toxicity of vinylidene chloride in mice and rats and its alteration by various treatments. J. Toxicol. Environ. Health Part A Curr. Issues.

[64] Posthuma A.R., Kraus J.G., Rutherford J.A. (1985). Cleanup of a Vinylidene Chloride and Phenol Spill. Management of Toxic and Hazardous Wastes.

[65] Sathishkumar K., Rangan V., Gao X., Uppu R.M. (2007). Methyl vinyl ketone induces apoptosis in murine GT1-7 hypothalamic neurons through glutathione depletion and the generation of reactive oxygen species. Free. Radic. Res..

[66] Sugawara H., Norimoto H., Zhou Z. (2022). Methyl vinyl ketone disrupts neuronal survival and axonal morphogenesis. J. Toxicol. Sci..

[67] National Center for Biotechnology Information PubChem Compound Summary for CID 6570, Methyl Vinyl Ketone. https://pubchem.ncbi.nlm.nih.gov/compound/Methyl-vinyl-ketone.

[68] Moore C.J. (2008). Synthetic polymers in the marine environment: A rapidly increasing, long-term threat. Environ. Res..

[69] Moroni M., Lupo E., La Marca F. (2019). Hydraulic separation of plastic wastes. Use of Recycled Plastics in Eco-Efficient Concrete.

[70] Atwood E.C., Falcieri F.M., Piehl S., Bochow M., Matthies M., Franke J., Carniel S., Sclavo M., Laforsch C., Siegert F. (2019). Coastal accumulation of microplastic particles emitted from the Po River, Northern Italy: Comparing remote sensing and hydrodynamic modelling with in situ sample collections. Mar. Pollut. Bull..

[71] Kazour M., Terki S., Rabhi K., Jemaa S., Khalaf G., Amara R. (2019). Sources of microplastics pollution in the marine environment: Importance of wastewater treatment plant and coastal landfill. Mar. Pollut. Bull..

[72] Birch G., Nath B., Chaudhuri P. (2015). Effectiveness of remediation of metal-contaminated mangrove sediments (Sydney estuary, Australia). Environ. Sci. Pollut. Res..

[73] Agoramoorthy G., Chen F.-A., Hsu M.J. (2008). Threat of heavy metal pollution in halophytic and mangrove plants of Tamil Nadu, India. Environ. Pollut..

[74] Einollahipeer F., Khammar S., Sabaghzadeh A. (2013). A study on heavy metal concentration in sediment and mangrove (*Avicenia marina*) tissues in Qeshm island, Persian Gulf. J. Nov. Appl. Sci..

[75] Arisekar U., Shakila R.J., Shalini R., Jeyasekaran G., Sivaraman B., Surya T. (2021). Heavy metal concentrations in the macroalgae, seagrasses, mangroves, and crabs collected from the Tuticorin coast (Hare Island), Gulf of Mannar, South India. Mar. Pollut. Bull..

[76] Harris A., Xanthos S.J., Galiotos J.K., Douvris C. (2018). Investigation of the metal content of sediments around the historically polluted Potomac River basin in Washington DC, United States by inductively coupled plasma-optical emission spectroscopy (ICP-OES). Microchem. J..

[77] Srikanth S., Kaihekulani S., Lum Y., Chen Z. (2015). Mangrove root: Adaptations and ecological importance. Trees.

[78] Chen T., Cai X., Wu X., Karahara I., Schreiber L., Lin J. (2011). Casparian strip development and its potential function in salt tolerance. Plant Signal. Behav..

[79] Rillig M.C., Lehmann A., de Souza Machado A.A., Yang G. (2019). Microplastic effects on plants. New Phytol..

[80] Yin L., Wen X., Huang D., Du C., Deng R., Zhou Z., Tao J., Li R., Zhou W., Wang Z. (2021). Interactions between microplastics/nanoplastics and vascular plants. Environ. Pollut..

[81] Roy T., Dey T.K., Jamal M. (2023). Microplastic/nanoplastic toxicity in plants: An imminent concern. Environ. Monit. Assess..

[82] Sun X.D., Yuan X.Z., Jia Y., Feng L.J., Zhu F.P., Dong S.S., Liu J., Kong X., Tian H., Duan J.L. (2020). Differentially charged nanoplastics demonstrate distinct accumulation in Arabidopsis thaliana. Nat. Nanotechnol..

[83] de Souza Machado A.A., Kloas W., Zarfl C., Hempel S., Rillig M.C. (2018). Microplastics as an emerging threat to terrestrial ecosystems. Glob. Change Biol..

[84] Machado A.A.S., Lau C.W., Till J., Kloas W., Lehmann A., Becker R., Rillig M.C. (2018). Impacts of microplastics on the soil biophysical environment. Environ. Sci. Technol..

[85] Helmberger M.S., Tiemann L.K., Grieshop M.J. (2020). Towards an ecology of soil microplastics. Funct. Ecol..

[86] Kannan N., Thirunavukkarasu N., Suresh A., Rajagopal K. (2016). Analysis of heavy metals accumulation in mangroves and associated mangroves species of Ennore mangrove ecosystem, east coast India. Indian J. Sci. Technol..

[87] Coppock R.L., Cole M., Lindeque P.K., Queirós A.M., Galloway T.S. (2017). A small-scale, portable method for extracting microplastics from marine sediments. Environ. Pollut..

[88] Van Ranst E., Verloo M., Demeyer A., Pauwels J.M. (1999). Manual for the Soil Chemistry and Fertility Laboratory: Analytical Methods for Soils and Plants Equipment, and Management of Consumables.

[89] Sujka K., Koczoń P. (2018). The application of FT-IR spectroscopy in discrimination of differently originated and aged whisky. Eur. Food Res. Technol..

