# Supplementary material for: Accumulation of Plastics and Trace Elements in the Mangrove Forests of Bima City Bay, Indonesia
Source: Plants (Basel). 2023 Jan 19;12(3):462. doi: 10.3390/plants12030462 (PMC9919253; doi:10.3390/plants12030462)
Supplement: Supplementary file 1 [file plants-12-00462-s001.zip › plants-2086613-supplementary.pdf]

Microplastic and trace elements accumulation in the mangrove forests of the Bima City Bay, Indonesia

Hanna Moniuszko [1], Win Ariga Mansur Malonga [1], Piotr Koczoń [2], Sofie Thijs [3], Robert Popek [1] and Arkadiusz Przybysz [1,*]

[1] Section of Basic Research in Horticulture, Department of Plant Protection, Institute of Horticultural Sciences, Warsaw University of Life Sciences – SGGW (WULS-SGGW), Nowoursynowska 159, 02-776 Warsaw, Poland
[2] Department of Chemistry, Institute of Horticultural Sciences, Warsaw University of Life Sciences – SGGW (WULS-SGGW), Nowoursynowska 159, 02-776 Warsaw, Poland
[3] Environmental Biology, Centre for Environmental Sciences, Hasselt University, Agoralaan Building D, 3590 Diepenbeek, Belgium

* Correspondence: arkadiusz_przybysz@sggw.edu.pl

Table S1. Experimental areas.

Number on map	Geographical coordinates	Description
Hotel area (Spot 1)	8°28'16.7"S 118°43'09.8"E	Located close to hotels and recreation areas for tourists; surrounded with coffee shops and restaurants; well maintained. The distance from the sampling point to the residential area is 0.030 km, to warehouse 0.065 km, and to hotels 0.30 Km.
Market area (Spot 2)	8°27'47.4"S 118°43'19.6"E	The biggest traditional market in Bima, mostly dirty and unorganized. Due to the scarcity of garbage cans merchants illegally throw waste into nearby mangrove forests. The distance from the sampling point to the traditional market is 0.084 km.
River mouth area (Spot 3)	8°27'31.2"S 118°42'40.6"E	The mouth of the largest river in the city of Bima. Most locals still throw garbage into the river; also sewage is discharged directly into the river. The distance from the sampling point to the river mouth is 0.050 km.
Port area (Spot 4)	8°27'06.5"S 118°43'05.1"E	The main port of Bima. The distance from the sampling point to the fisherman’s house is 0.030 km, to the resident port (illegal port) is 0.083 km, and to Bima Port is 0.38 km.
Rural area (Spot 5)	8°31'03.8"S 118°40'05.4"E	The rural spot; located considerably far from the city center (31 km). There is a small village (with no industrial activities) and salt ponds nearby. The distance from the sampling point to the several sites including salt ponds (0.14 km) and village settlements (0.5 km).
